# m^6^A mRNA Methylation Analysis Provides Novel Insights into Pigmentation in Sheep Skin

**DOI:** 10.1080/15592294.2023.2230662

**Published:** 2023-06-30

**Authors:** Yuanyuan Zhao, Jinzhu Meng, Xingchao Song, Qingming An

**Affiliations:** a Guizhou Provincial Key Laboratory for Biodiversity Conservation and Utilization in the Fanjing Mountain Region, Tongren University, Tongren, Guizhou, P. R. China; b College of Veterinary Medicine, Hunan Agricultural University, Changsha, Hunan, P.R. China

**Keywords:** Sheep, m^6^A mRNA methylation, Pigmentation, MeRIP-seq

## Abstract

N6-methyladenosine (m^6^A) is the most universal post-transcriptional modification of mRNA which may play important roles in verious species. However, the potential roles of m^6^A in the pigmentation of skin are not completely understood. To explore the role of m6A modification in pigmentation of sheep skin, we used MeRIP-seq and RNA-seq to profile the skin transcriptome in black and white coat color (*n=*3). Our results showed that an average of 7701 m^6^A peaks were obtained for all samples and the average length was 305.89 bp. The GGACUU sequence was the most enrichment motif and shared in black skin and white skin. The m^6^A peaks were mainly enriched in the CDS, 3'UTR and 5'UTR, especially in CDS region near the stop codon of the transcript. 235 signiﬁcantly differential peaks were found in black skin vs. white skin. The KEGG signaling pathways of downregulated and upregulated m^6^A peaks were mainly enriched in AGE-RAGE signaling pathway in diabetic complications, Viral carcinogenesis, Transcriptional misregulation in cancer, ABC transporters, Basal transcription factors and Thyroid hormone synthesis (*P* value <0.05). For RNA-seq, 71 differently expressed genes (DEGs) were scanned in black skin vs. white skin. DEGs were significantly enriched in tyrosine metabolism, melanogenesis, neuroactive ligand-receptor interaction pathway (*P* value <0.05). Combined m^6^A-seq and RNA-seq analysis showed that the hyper-up genes and hypo-up genes were both enriched in ErbB signaling pathway (*P* value <0.05). In conclusion, it provide a basis for further research into the functions of m^6^A methylation modiﬁcations in pigmentation.

## Introduction

Coat colour is a direct reflection of pigmentation in fleece-producing animals. In mammals, the main pigments of the skin and hair are melanins, which are synthesized in melanosomes of melanocytes and then secreted into keratinocytes [1]. Multiple genes and signalling pathways involves in melanin synthesis in mammals, such as tyrosinase (encoded by *TYR*) catalyse the oxidation of tyrosine (or of L-3,4-dihydroxyphenyla-lanine [L-dopa]) to dopaquinone, which is the initial reaction in melanin synthesis [2]. *TYRP1* (TYR-related protein 1) and *DCT*/*TYRP2* (dopachrome tautomerase) modulate the eumelanin synthesis [3,4]. Pre-melanosome protein (encoded by *PMEL*) is the component of the intraluminal fibrous sheets on which eumelanins are deposited in melanosomes [5]. *SLC45A2* directly impact the pH of maturing melanosomes [6].

Epigenetics refers to the underlying genetic changes that affect gene expression without altering the original sequence of DNA nucleotides. DNA methylation, histone acetylation and deacetylation, transcription factor, microRNAs (miRNAs), long noncoding RNAs (lncRNAs), circular RNAs (circRNAs) and interactions have been shown to regulates melanogenesis [7]. However, how RNA methylation regulating pigmentation in skin is unclear. N6-methyladenosine (m^6^A), discovered in messenger RNA (mRNA) in 1974 from rat, is the most universal post-transcriptional modification of mRNA from bacteria, viruses, yeast, fruitflies, plants and mammals [8–12]. The m^6^A methylation is catalysed by methyltransferases (METTL3, METTL14, METTL16, CAPAM, METTL5, TRMT11, ZCCHC4, WTAP), removed by demethylases (FTO, ALKBH5) and recognized by reader proteins (YTHDF1/2/3,) [13,14]. M^6^A modifications, regulating the stability, splicing, translation, and degradation of mRNAs, may play important roles in growth, reproduction, nerve development, fat metabolism, immune responses, tumour invasion and other physiological processes [15–17]. Previous study showed that compared with melanocytes, the expression level of *METTL3* in melanoma cell lines was higher and play a role in invasion/migration [16]. *METTL14* modulates retinal pigment epithelial (RPE) activity [18]. *METTL3* attenuates epithelial-mesenchymal transition, proliferative vitreoretinopathy and high-glucose induced pyroptosis of retinal pigment epithelial cells [19,20]. However, the role of m^6^A modifications in pigmentation has not known.

To explore the role of m^6^A modification in pigmentation of sheep skin, we performed N6-methyladenosine sequencing (m^6^A-seq) and RNA sequencing (RNA-seq) to investigate differentially methylated genes (DMGs) and differentially expressed genes (DEGs) in black and white skin of sheep. Our results provide a theoretical basis for further research into the molecular mechanisms of m^6^A modiﬁcation in pigmentation.

## Materials and methods

All related experiments involving sheep were conducted in strict compliance with relevant guidelines set by the Ethics Committee of Tongren University, China (Approval ID: TREDU2022–016).

## Animals and sample collection

Small Tailed Han Sheep used in this study were raised under the same conditions at Taigu Haihong Animal Husbandry Co. LTD. (Taigu, China). Three one-year-old sheep similar in size with black-and-white coat colour were selected. The black hair and adjacent white hair were cut off, and the black skin and adjacent white skin were taken using skin biopsy borer with a diameter of 1 cm. Four skin pieces of each colour were taken from each sheep. All samples were put into 1.5 mL centrifuge tubes, labelled and stored in liquid nitrogen for RNA extraction.

## RNA isolation and fragmentation

Total RNA from 6 samples (1 black and 1 white skin tissue each sheep) was extracted using TRlzol™ Reagent (Thermo Fisher Scientifi, MA, USA) according to the manufacturer’s instructions and genomic DNA was removed with DNase I (Roche Diagnostics, IN, USA). A260/A280 > 1.9, A260/A230 > 1.7 was used to evaluate RNA purity using a NanoDrop ND-1000 instrument (NanoDrop, DE, USA). The concentration of total RNA was measured by Qubit RNA HS Assay Kit (Thermo Fisher Scientific, MA, USA). The purified mRNA (25 μg) was randomly broken into ~150 nt fragments using RNA Fragmentation Buffer (100 mM TrisHCl, 100 mM ZnCl_2_) at 70°C for 6 min. 98% of the fragmented mRNA was used for immunoprecipitation (IP), and the rest (2%) for IP control (Input).

## RNA immunoprecipitation

m^6^A MeRIP is based on the previously described m^6^A-seq protocol [21] with several modifications: 30 μL of protein A magnetic beads (Thermo Fisher Scientific, MA, USA) were washed twice by IP buffer (150 mM NaCl, 10 mM Tris-HCl [pH 7.5], 0.1% IGEPAL CA-630), resuspended in 500 μL of IP buffer, and tumbled with 5 μg anti-m^6^A antibody (Synaptic Systems, Gottingen, Germany) at 4°C for 6 h. It was then incubated in 500 μL IP buffer with 5 μL of RNasin Plus RNase Inhibitor (40 U/μL, Promega, WI, USA) at 4°C for 2 h. After extensive washing, the m^6^A-enriched fragmented RNA was eluted by 200 μL of RLT buffer supplied in RNeasy Mini Kit (QIAGEN, Dusseldorf, Germany) for 2 min at room temperature. Thereafter, supernatant was collected and 400 μL of 100% ethanol was added, then m^6^A-enriched RNA was purified using an RNeasy MiniElute spin column (QIAGEN, Dusseldorf, Germany). Finally, The m^6^A-enriched RNA was eluted with 14 μL ultrapure H_2_O.

## M^6^A library preparation and sequencing

The amount of 2 μL eluted RNA and input RNA was reverse transcribed with High-Capacity cDNA Reverse Transcription Kit (Thermo Fisher Scientific, MA, USA). Transcriptome-wide interrogation was pursued by deep sequencing using SMARTer Stranded Total RNA-Seq Kit version 2 (Pico Input Mammalian, Takara/Clontech, Osaka, Japan) on Illumina Novaseq 6000 platform. 150 bp paired-end reads were generated.

## RNA-seq

A total amount of 1 µg RNA per sample was used as input material for the RNA sample preparations. Sequencing libraries were generated using NEBNext® UltraTM RNA Library Prep Kit for Illumina® (NEB, USA) following manufacturer’s recommendations and index codes were added to attribute sequences to each sample.

## Bioinformatic analysis of m^6^A-seq

Trimmomatic software (v0.32) were used to remove adapter and low quality reads [22]. Quality distribution plot and base content distribution were generated by FastQC (http://www.bioinformatics.babraham.ac.uk/projects/fastqc/). The software STAR was used to align reads to the sheep reference genome (Oar_v4.0, https://www.ncbi.nlm.nih.gov/assembly/GCF_000298735.2/). Uniquely mapped reads were used for subsequent analysis. The R software package MetPeak (Cutoff threshold: PEAK_CUTOFF_*P* value = 0.05, FOLD_ENRICHMENT = 1) was used to call peak and IGV software (http://www.igv.org) were used to visualize. The R software package Guitar was used to calculate the densty plot of peaks in 3‘UTR, CDS and 5‘UTR. ChIPseeker (https://bioconductor.org/packages/ChIPseeker) and HOMER (http://homer.ucsd.edu/homer/motif) softwares were used to annotate the peaks and perform motif analysis, respectively. The MeTDiff software was used to analyse the difference the MeRIP-seq data between black and white skin (*P* value < 0.05 and Log_2_FC >1 as upregulated peak, *P* value < 0.05 and Log2^FC^ <-1 as downregulated peak). Gene Ontology (GO) and Kyoto Encyclopedia of Genes and Genomes (KEGG) analyses of m^6^A modified genes (The 3‘UTR of differentially methylated mRNA was modified by m^6^A) were performed by the DAVID database (http://david.abcc.ncifcrf.gov/) and the KEGG database (https://www.kegg.jp/), respectively.

## Bioinformatic analysis of RNA-seq

Raw data (raw reads) of fastq format were firstly processed through in-house perl scripts to obtain clean data (clean reads), which were used for further analysis. STAR was used to align clean reads to reference genome. HTSeq v0.6.0 was used to count the reads numbers mapped to each gene. And then FPKM (expected number of Fragments Per Kilobase of transcript sequence per Millions base pairs) was calculated based on the length and reads count of genes. The differentially expressed genes (DEGs) were filtered by DESeq2 algorithm with the criteria of |log_2_FC| > 1 and *P* value < 0.05. Gene Ontology (GO) and Kyoto Encyclopedia of Genes and Genomes (KEGG) analyses of differentially expressed genes were performed by the DAVID database (http://david.abcc.ncifcrf.gov/) and the KEGG database (https://www.kegg.jp/), respectively. Fisher’s exact test was applied to identify the significant GO categories, significant pathway and FDR was used to correct the *P* values.

## Real-Time quantitative PCR (RT-Qpcr)) and m^6^A IP (MeRIP) followed RT-Qpcr (MeRIP-Qpcr)

IP RNA and Input RNA (1 μg) from each sample were synthesized cDNA by PrimeScript™ RT Master Mix (Perfect Real Time) (TAKARA, Dalian, China). Thereafter, a TB Green® Fast qPCR Mix (TAKARA, Dalian, China) and a LightCycler 480II (Roche, Basel, Switzerland) were used to perform RT-qPCR and MeRIP-qPCR. The primers are presented in Table 1, β-actin served as internal control. The relative expression of genes were determined by the 2 ^– ΔΔCt^.

**Table 1.** Primer sequences for RT-Qpcr and MeRIP-Qpcr..

Genes	Primer sequence (5’−3’)	Product length (bp)	Accession NO.
*CSPG4*	F: CTGGTCCGGCACAAGAAGAT R: AGAACACAATGTCCGCTGGT	109	XM_027957164.2
*MAML2*	F: GGTTTAACCTCGCCACTCCA R: CCCTTACTTCGGACACTGGT	105	XM_015100757.3
*DENND2B*	F: CCAGAGCCTCATGGTTCCAG R: TCTTGTTGGCAGTCATGGTCA	108	XM_042233083.1
*ADAMTS1*	F: CGACAAATGTGGCGTCTGTG R: TTTGTGGCTCCGGTTGGAAT	117	XM_004002797.5
*MAP1B*	F: CTACGTGGTGAGTGGGAACG R: ATGAGTCGGGATCAGGGTCA	121	XM_027980019.2
*GAB2*	F: AGAGACAGTGCCTACGACCT R: GCTGGGCGTCTTGAAGGTAT	114	XM_042237732.1
*TYRP1*	F: TGGCCAGGTGAGTACTGAAA R: CAGAATGGGGTCCCGACAAA	190	EU760771.1
*SLC45A2*	F: CTCTGGCCATGTGCACCTTA R: GAGAGCCACAAAGCAACAGC	296	XM_004017064.4
*TYR*	F: GCACAACCGGGAATCCTACA R: CCAGCACAGCAGTAAGGACA	221	NM_001130027.1
*TCL1A*	F: CCAACCCTGTGTGATCTGCT R: GTATGAGGACCCCGAAGCTG	120	XM_012099174.4
*ZAR1*	F:GGCACTAACAAGAATTGTAAACAGA R: TTGCCAAACAGCCTTTCACG	303	XM_042251401.1
*MCP-3*	F: CACACCATCACGGACCAAGA R: CACTGCACATCATCCCTGGT	202	NM_001009411.2
*β-actin*	F: GCAGGAGTACGATGAGTCCG R: AACCGACTGCTGTCCCCTT	238	DQ152927.1

## Results

### MeRIP-seq summary

To explore the role of m^6^A modification in pigmentation of sheep skin, the black and white skins of three sheep were collected for m^6^A sequencing. The IP libraries (MeRIP-seq) and the input libraries (RNA-seq) of black skin and white skin were constructed. As a result, a total of 56,239,292–58,047,254 and 61,572,416–65,348,920 raw reads in the IP libraries and in the input libraries were obtained for black skin, respectively. Meanwhile, a total of 53,302,052–59,653,724 and 55,037,238–64,703,960 raw reads in the IP libraries and in the input libraries were obtained for white skin, respectively. An average of more than 50,143,426 clean reads per sample were obtained. The Q20 and Q30 values were at least 97.15% and 91.74% for all sample, respectively (Table 2). In addition, more than 50% clean reads were aligned to the sheep reference genome. At least 18,019,944 clean reads were uniquely mapped and the percent of uniquely mapped reads >70% for all samples (Supplementary Table S1).

**Table 2.** Sequence statistics and quality control.

Sample Name	Raw Reads	Clean Reads	Clean Reads (%)	Q20%)	Q30%)	GC Content (%)
BLACK1-IP	56239292	53025640	88.98	97.47	92.58	53.8
BLACK1-input	65348920	55488324	75.71	97.82	93.51	57.61
BLACK2-IP	57702866	53712660	87.41	97.15	91.75	52.29
BLACK2-input	61572416	53491488	76.40	97.87	93.6	54.53
BLACK3-IP	58047254	53967086	87.495	97.11	91.74	51.59
BLACK3-input	66534292	54903570	73.85	97.78	93.37	57.79
WHITE1-IP	56076312	52831282	88.35	97.65	92.99	54.35
WHITE1-input	64703960	55491504	73.12	98.06	94.1	55.79
WHITE2-IP	59653724	56942474	88.93855	97.74	93.28	53.88
WHITE2-input	55037238	50143426	81.96	97.86	93.57	56.9
WHITE3-IP	53302052	50733478	89.88	97.83	93.54	51.93
WHITE3-input	58191410	52316806	81.33	97.97	93.86	57.66

## General features of sheep m^6^A methylation

**Figure 1.**
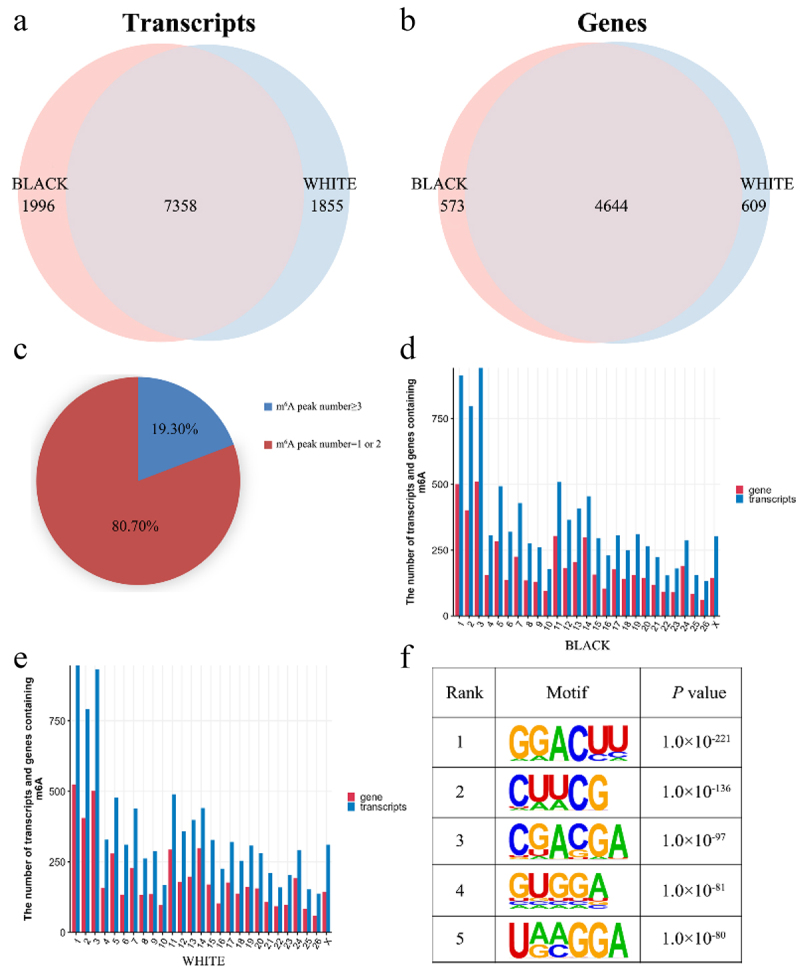
Peak Distribution. (a) m^6^A peaks distributions in black and white skin of sheep among mRNA transcripts. (b) m^6^A peaks distributions in black and white skin of sheep among genes. (c) Distributions of differential m^6^A peaks in per gene. (d) Numbers of m^6^A peaks in the mRNA transcripts of chromosomes in black skin of sheep. (e) Numbers of m^6^A peaks in the mRNA transcripts of chromosomes in white skin of sheep. (f) the top five motif sequences for m^6^A-containing peaks from all samples.

An average of 7701 peaks were obtained for all samples and the average length was 305.89 bp (Supplementary Table S2, S3). After merging the three replicates, 9354 m^6^A modified peaks and 5217 genes were found in black skin. Meanwhile, 9213 m^6^A modified peaks and 5253 genes were detected in white skin (Figures 1a, 1b). The count of m^6^A peaks in one modified gene was in the range of 1 to 30, and the average was 1.2 to 1.32 m^6^A peaks in six samples. Meanwhile, 80.70% of the modified genes had only one or two m^6^A peaks, the remaining genes (19.30%) contained three or more peaks in sheep skin (Figure 1c). Furthermore, we analysed the distributions of peaks on the sheep chromosomes in skin. Interestingly, there were more peaks on chromosomes 1, 2, 3, 5, 11, and 14 than on other chromosomes, and the most peaks were distributed on chromosome 1 in black skin and chromosome 3 in white skin (Figure 1d,e). To identify RRACH (R, purine; A, m^6^A; H, non-guanine base) motif of sheep skin, HOMER software was used. The results showed that more than 15 motifs are identified in each sample and the five motifs with the smallest *P* value from each group were used for subsequent analysis (Figure 1f). The GGACUU sequence was shared in black and white skin, which inversely complemented two miRNA (hsa-miR-302e and hsa-miR-2114) seed sequences.

## Topological pattern of sheep m^6^A methylation

**Figure 2.**
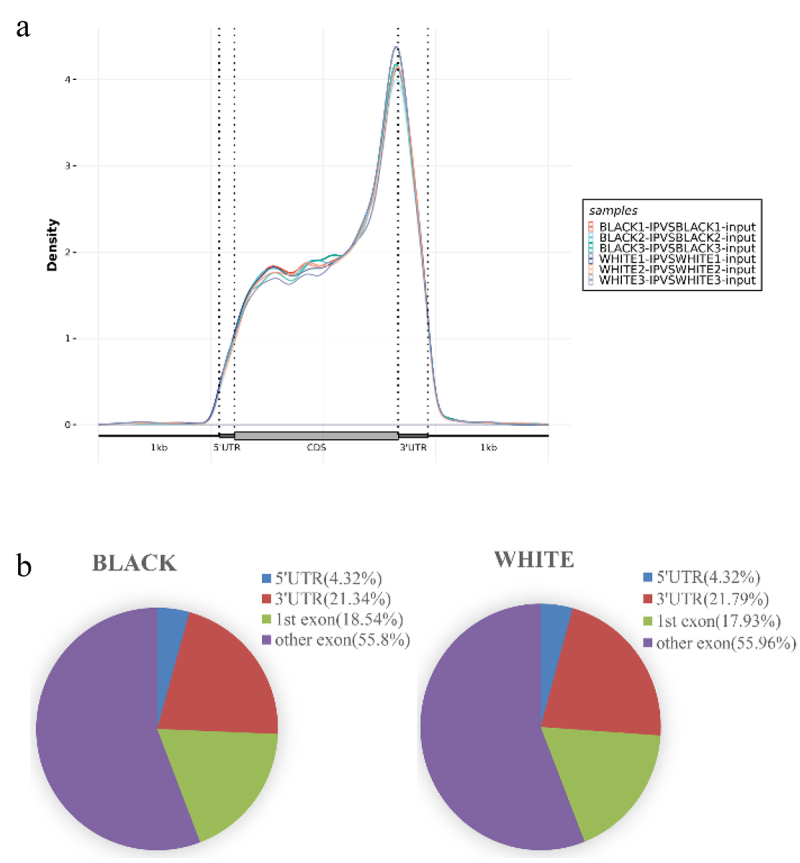
Peak Distribution. (a) Density distributions of m^6^A peaks in different gene functional elements (5'UTR, CDS, and 3'UTR) in each sample. (b) Distributions of m^6^A peaks in different gene functional elements (5'UTR, 3'UTR, 1 st exon, and other exons) in black skin and white skin.

The distributions of peaks in gene functional elements were analysed. As a result, the m^6^A peaks in black skin and white skin were mainly enriched in the CDS, 3‘UTR and 5‘UTR, especially in CDS region near the stop codon of the transcript (Figure 2a). To further evaluate the distribution of m^6^A modified peak, the transcript was divided into the 5‘UTR, 3‘UTR, 1st exon and other exon. As a result, the highest enrichment of m^6^A modified peaks was in other exon, followed by 3‘UTR, 1st exon and 5‘UTR (Figure 2b).

## Differential m^6^A peaks between black and white skin

**Figure 3.**
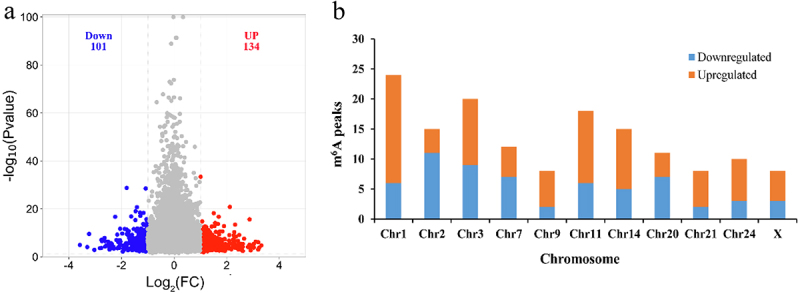
m^6^A peaks in black skin and white skin of sheep. (a) Signiﬁcantly different m^6^A peaks in black skin vs. white skin. (b) Signiﬁcant differences in the distributions of m^6^A peaks on sheep chromosomes.

MeTDiff software was used to analyse differential m^6^A peaks between black skin and white skin based on *P* value < 0.05 and | Log2^FC^ |>1. The result showed that 235 differential peaks and 226 genes (differentially methylated genes, DMGs) were scanned, of which 134 signiﬁcantly upregulated peaks in 127 genes and 101 signiﬁcantly downregulated peaks in 99 genes were found in black skin vs. white skin (Figure 3a and Supplementary Table S4). The top 10 upregulated genes and downregulated genes are present (Table 3).

**Table 3.** Top 10 signiﬁcantly upregulated and downregulated m^6^A peaks (BLACK vs. WHITE).

Chromosome	ChromStart	ChromEnd	Gene name	*P* value	Strand	Log_2_FC	Regulation	Peak region
4	23383792	23384256	*ARL4A*	1×10^−13^	+	−3.94	down	CDS
21	19123523	19125178	*TENM4*	7.9×10^−21^	+	−3.94	down	CDS
18	31064789	31065785	*CSPG4*	6.3×10^−12^	+	−3.70	down	CDS
15	15665020	15666219	*MAML2*	6.3×10^−16^	+	−3.63	down	3‘UTR
15	46957631	46983543	*DENND2B*	7.9×10^−17^	+	−3.62	down	CDS
5	40849569	40849904	*TRIM52*	1.3×10^−11^	-	−3.62	down	CDS
2	2.24E+08	2.24E+08	*PLEKHM3*	7.9×10^−15^	-	−3.43	down	5’ UTR
23	53963974	53964273	*CTIF*	5×10^−14^	+	−3.43	down	3’ UTR
16	4376992	4379027	*SH3PXD2B*	5×10^−13^	-	−3.38	down	3’ UTR
18	68897002	68898246	*BAG5*	4.9×10^−10^	-	−3.36	down	CDS
11	9176017	9189954	*TNRC6C*	2. ×10^−19^	+	3.95	up	CDS
14	52705416	52706353	*ITPKC*	5×10^−13^	-	3.86	up	-
21	42866241	42867012	*UQCC3*	6.3×10^−14^	+	3.72	up	3’ UTR
13	25201358	25202258	*OTUD1*	2×10^−21^	+	3.67	up	CDS
11	27286934	27287133	*ANKRD40*	2.5×10^−11^	-	3.62	up	3’ UTR
14	54169492	54170493	*CIC*	1.4×10^−10^	-	3.60	up	CDS
21	10853343	10854221	*CREBZF*	1.1×10^−8^	+	3.57	up	CDS
18	47442175	47443157	*CLEC14A*	1.7×10^−9^	-	3.53	up	CDS
16	74060446	74061095	*ICE1*	1×10^−15^	-	3.51	up	CDS
3	5933126	5933674	*ABL1*	3.2×10^−12^	-	3.49	up	CDS

The distribution of differential m^6^A peaks on chromosomes showed that the downregulated peaks were most enriched on chromosome 2 (11 differential peaks), while the upregulated peaks were most enriched on chromosome 1 (18 differential peaks). The number of the downregulated peaks and upregulated peaks on chromosome 1 were 6 and 18 was most different (Figure 3B). The distribution of differential peaks in genes was counted. Only two genes (*CSPG4*, *MAML3*) contained two downregulated peaks, but five genes (*ADAMTS1*, *COL6A2*, *GAB2*, *MAP1B*, *TNRC18*) contained two upregulated peaks. Even *PLEC* gene owned 3 upregulated peaks. The remaining genes (97.98% downregulated genes and 95.28% upregulated genes) all have a single differential peak (Supplementary Table S4).

## GO and KEGG analysis of genes presenting differential m^6^A Peaks

**Figure 4.**
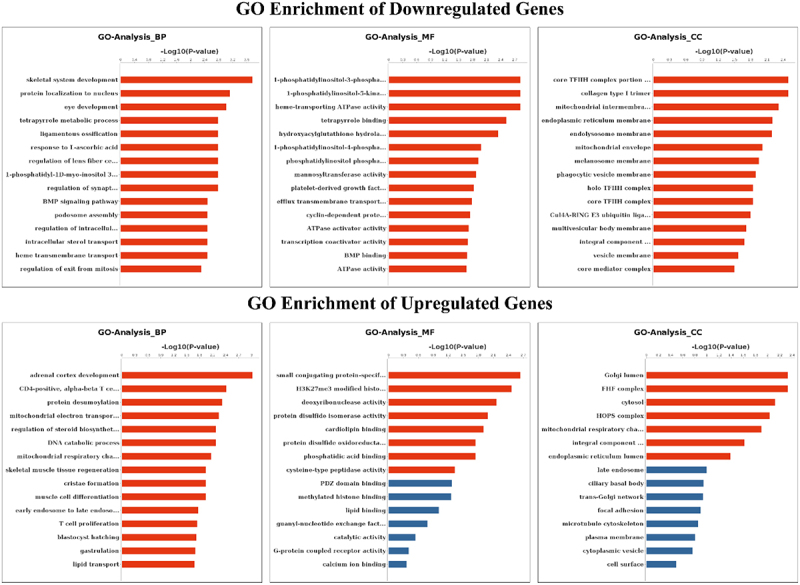
GO analyses of m^6^A modified genes. Top 15 GO terms of the differentially methylated downregulated genes and upregulated genes in three categories (BP, MF, CC). The red column indicates significant enrichment, while the blue column indicates insignificant enrichment.

**Figure 5.**
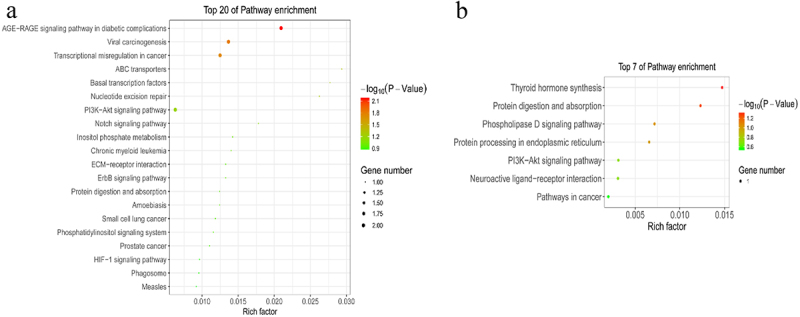
KEGG analyses of m^6^A modified genes. The enriched KEGG pathways of the differentially methylated downregulated genes (a) and upregulated genes (b).

To explore the role of m^6^A modification in sheep skin and its relationship with pigmentation, the functions of m^6^A modified genes were analysed using the DAVID database and the KEGG database. According to GO terms, the functions were divided into three categories: the biological process (BP), cellular component (CC), and molecular function (MF) categories. Downregulated m^6^A peaks were significantly enriched in 97 BP, 18 CC and 21 MF GO terms, and Upregulated m^6^A peaks were significantly enriched in 21 BP, 7 CC and 8 MF GO terms, the top 15 of three categories were showed in Figure 4. GO terms related to pigmentation involve in regulation of synaptic transmission, dopaminergic, melanosome assembly, regulation of dopamine metabolic process, melanosome organization, negative regulation of kinase activity and melanosome membrane. The KEGG signalling pathways of downregulated m^6^A peaks were mainly enriched in AGE-RAGE signalling pathway in diabetic complications, Viral carcinogenesis, Transcriptional misregulation in cancer, ABC transporters, Basal transcription factors (*P* value < 0.05). Meanwhile, the KEGG signalling pathways of upregulated m^6^A peaks were mainly enriched in Thyroid hormone synthesis (*P* value < 0.05) (Figure 5).

## Differently expressed genes (RNA-seq) in black and white skin

**Figure 6.**
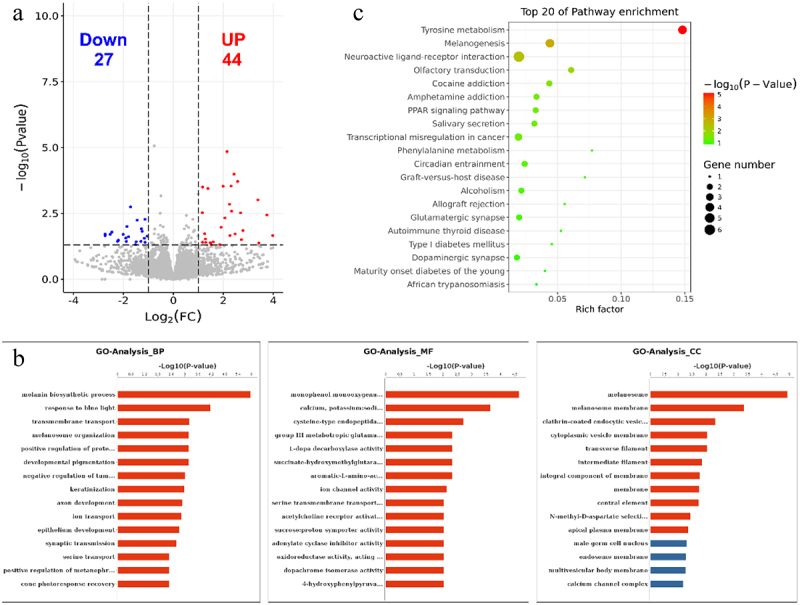
GO and KEGG analyses of DEGs. (a) Volcano plot showing the differential gene expression in black skin vs. white skin of sheep. (b) Top 20 KEGG pathways enriched for DEGs. (c) Top 15 GO terms of DEGs in three categories (BP, MF, CC). The red column indicates significant enrichment, while the blue column indicates insignificant enrichment.

The RNA-seq data for each sample were used to analyse differently expressed genes between black skin and white skin. A total of 71 DEGs were scanned, among which 27 DEGs were downregulated and 44 DEGs were upregulated in black skin vs. white skin (Figure 6a, Supplementary Table S5). The top 10 upregulated genes and downregulated genes are listed in Table 4. The functions of these DEGs were analysed and the GO enrichment and KEGG pathway were evaluated. As a result, the top 15 GO terms of biological process (BP), cellular component (CC) and molecular function (MF) were exhibited in Figure 6b. The DEGs were significantly enriched in melanin biosynthetic process, response to blue light, transmembrane transport, melanosome organization, positive regulation of protein kinase A signalling, developmental pigmentation, melanosome and L-dopa decarboxylase activity, which were closely related to pigmentation of skin. KEGG analysis showed that DEGs were significantly enriched in Tyrosine metabolism (4 DEGs), Melanogenesis (4 DEGs), Neuroactive ligand–receptor interaction (6 DEGs), Neuroactive ligand–receptor interaction Olfactory transduction (2 DEGs), Cocaine addiction (2 DEGs), Amphetamine addiction (2 DEGs), PPAR signalling pathway (2 DEGs), Salivary secretion (2 DEGs), Transcriptional misregulation in cancer (3 DEGs) (*P* value < 0.05). Top 20 enriched KEGG pathways are listed (Figure 6c).

**Table 4.** Top 10 signiﬁcantly upregulated and downregulated genes (BLACK vs. WHITE).

Locus	Gene name	*P* value	Strand	Log_2_FC	Regulation
chr18:60855163–60858729	*TCL1A*	0.034	-	−4.55759	down
chr 6:73764050–73767864	*ZAR1*	0.026	+	−4.5438	down
chr 18:32645618–32716451	*MCP-3*	6.37×10^−7^	+	−4.12923	down
chr 7:75675023–75677892	*SIX1*	0.008	-	−4.117	down
-	*5S_rRNA*	0.022	-	−2.73541	down
chr 1:97071826–97207889	*SYCP1*	0.019	+	−2.73325	down
chr 1:219010098–219035477	*KNG1*	0.019	-	−2.53527	down
chr 3:146247496–146250747	*AQP5*	0.016	-	−2.45983	down
chr 14:56880795–56886640	*FOXA3*	0.036	+	−2.2333	down
chr 3:39631341–39658950	*CAPN14*	0.033	-	−2.20124	down
chr 2:87540480–87556594	*TYRP1*	6.9×10^−14^	+	13.08397	up
chr 16:41978919–42013172	*SLC45A2*	3.57×10^−11^	+	9.966668	up
chr 11:11951667–11954992	*TSPAN10*	1.68×10^−10^	+	9.599748	up
chr 2:79584125–79594791	*MLANA*	7.04×10^−12^	+	8.412334	up
chr 18:25349881–25450837	*TRPM1*	1.91×10^−7^	-	7.728241	up
chr 3:175255140–175263368	*PMEL*	2.16×10^−16^	+	6.455803	up
chr 21:7263507–7379923	*TYR*	2.33×10^−11^	-	5.898827	up
chr 5:5460092–5524649	*UNC13A*	0.004	+	5.244571	up
chr 8:12050576–12225332	*SNAP91*	0.0008	-	5.193136	up
chr 1:8353926–8356212	*KCNJ13*	0.006	+	4.950321	up

## Combined m^6^A-seq and RNA-seq analysis

**Figure 7.**
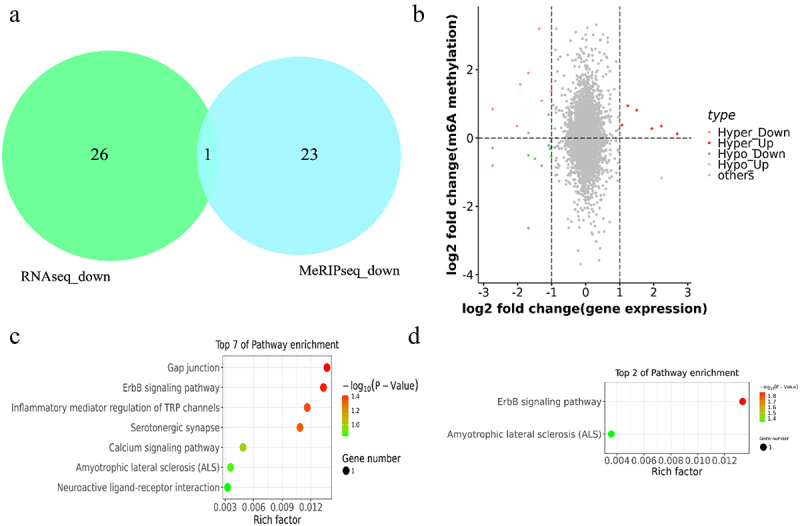
Combined m^6^A-seq and RNA-seq analysis. (a) the overlap gene between downregulated m^6^A methylation and downregulated mRNA in black skin vs. white skin (b) Four-quadrant diagram depicting the distributions of m^6^A modified genes and DEGs. (c-d) the enriched KEGG pathways among the identiﬁed (c) hyper-up genes and (d) hypo-up genes.

To further determine the effect of m^6^A modiﬁcation on pigmentation in sheep skin, the relationship of m^6^A modified genes and DEGs were analysed according to m^6^A-seq data and RNA-seq data. As a result, *BIIIB4* was the unique overlap gene which was downregulated in both m^6^A methylation and mRNA expression level in black skin vs white skin in one of the three sheep (Figure 7a). In addition, With |log_2_FC|>0 as the dividing line of m^6^A methylation and |log_2_FC|>1 as the threshold of mRNA differential expression, a total of 27 genes were divided into four groups: 9 hypermethylated and downregulated genes (hyper-down genes), 6 hypermethylated and upregulated genes (hyper-up genes), 11 hypomethylated and downregulated genes (hypo-down genes), 1 hypomethylated and upregulated genes (hypo-up genes) (Figure 7b and Supplementary Table S6). All genes were used for function analysis using KEGG database, which revealed that the hyper-up genes were mainly enriched in Gap junction, ErbB signalling pathway, Inflammatory mediator regulation of TRP channels and Serotonergic synapse (*P* value < 0.05) (Figure 7c). In addition, the hypo-up genes were mainly related to ErbB signalling pathway and Amyotrophic lateral sclerosis (ALS) (*P* value < 0.05) (Figure 7d). However, the hyper-down genes and hypo-down genes were not significantly enriched in any pathway (*P* value > 0.05)

## Validation of DEGs and DMGs by qPCR and MeRIP-Qpcr

**Figure 8.**
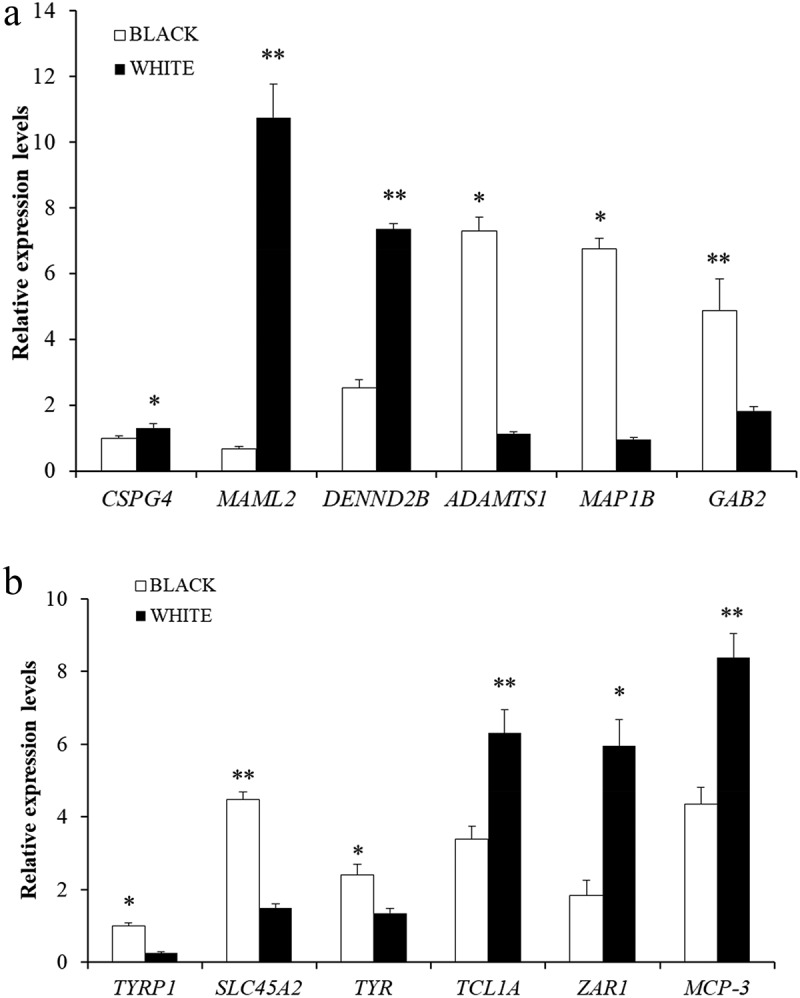
Veriﬁcation of DEGs and DMGs. (a) the m^6^A methylation modiﬁcations of six genes veriﬁed by MeRIP-Qpcr.. (b) the expression levels of six genes veriﬁed by qPCR.

To verify the MeRIP-Seq and RNA-seq sequencing data, we selected six DMGs (*CSPG4*, *MAML2*, *DENND2B*, *ADAMTS1*, *MAP1B*, *GAB2*) and six DEGs (*TYRP1*, *SLC45A2*, *TYR*, *TCL1A*, *ZAR1*, *MCP-3*) randomly and detected their expressions by MeRIP-qPCR and RT-qPCR. The result showed that the expression trends of these genes were consistent with the RNA-seq and m^6^A-seq (Figure 8a,b), which conﬁrmed the accuracy of m^6^A-seq and RNA-seq experiment.

## Discussion

Coat colour is an important trait in sheep. Coat colour is determined by the content and composition of melanin. Melanin produced in melanocytes can be divided into two types, eumelanin, which appears black to brown and pheomelanin which appears yellow to reddish brown. The genetic basis and many genes controlling coat colour of sheep has been found, such as melanocortin-1 receptor (*MC1R*), agouti signalling protein (*ASIP*), *TYR*, *TYRP1* and microphthalmia transcription factor (*MITF*) [23].

M^6^A modifications, regulating the stability, splicing, translation, and degradation of mRNAs, may play important roles in growth, reproduction, nerve development, fat metabolism, immune responses, tumour invasion and other physiological processes [15,16]. However, the function of m^6^A modifications on pigmentation is unclear. In this study, the m^6^A modifications of black skin and white skin of sheep were detected, 7358 peaks that were in common in the black skin and white skin, which accounted for 79.9% of all the peaks. This means that most m^6^A modifications are designed to maintain cellular metabolism. The average number of m^6^A peaks per gene was 1.2 to 1.32 in six samples, which is similar to that in pig liver (1.33–1.42) and mouse liver (1.34) [24,25]. The consensus sequences ‘RRACH’ were conserved in various species [26], which was also verified in sheep. The GGACUU sequence was the most enriched m^6^A motif in all samples. The distributions of peaks in gene functional elements were analysed. As a result, the m^6^A peaks in black skin and white skin of sheep were mainly enriched in the CDS, 3‘UTR and 5‘UTR, especially in CDS region near the stop codon of the transcript. The result agrees with that in human, mouse, pig and sheep liver tissue [14,15,25]. The stability, localization, expression, and translation of mRNA were regulated by 3'UTR where multiple RNA-binding proteins bind to plays a regulatory role and protein interaction [27].

Differential m^6^A peaks between black skin and white skin were analysed. The result showed that 235 differential peaks and 226 genes were scanned, of which 134 signiﬁcantly upregulated peaks in 127 genes and 101 signiﬁcantly downregulated peaks in 99 genes were found in black skin vs. white skin. Eight DMGs contains two or more peaks. Chondroitin sulphate proteoglycan 4 (*CSPG4*) is essential to the survival and growth of melanoma tumours by enhancing growth factor receptor-regulated pathways, such as sustained activation of ERK 1,2, and modulating integrin function [28]. ERK pathway was the key intracellular signalling pathway, which was involved in melanogenesis by the activation of signal transduction [29]. *ADAMTS1*(A Disintegrin-like And Metalloprotease domain with ThromboSpondin type I motifs) is upregulated in black skin compared with white skin is in accord with previous study. *ADAMTS* genes (*ADAMTS1*, *ADAMTS6* and *ADAMTS9*) may associate with age-related macular degeneration and *MAP1B* was higher expression in the macula of human eye [30,31]. *GAB2* amplification is critical for melanomas arising from sun-protected sites and in mast cell development *GAB2* is required for KitL/c-Kit signalling which is an important signal in melanogenesis [32–34]. Mastermind-like 3 (*MAML3*) are potential therapeutic targets for small cell lung cancer and pancreatic cancer [35]. Collagen VI (*COL6A1*, *COL6A2* and *COL6A3*) mutations result in disorders abnormal skin findings [36]. But there is no evidence that *MAML3*, *COL6A2*, *TNRC18*, *PLEC* are related to pigmentation. This study provides a new direction for these genes. Transcriptome of each sample were detected and a total of 71 DEGs were scanned, among which 27 DEGs were downregulated and 44 DEGs were upregulated in black skin vs. white skin. Upregulated genes *TYR*, *TSPAN10*, *TRPM1*, *MLANA*, *KCNJ13*, *TYRP1*, *DCT*, *SLC45A2, SLC24A5, MC1R* and *PMEL* have been proven to participate in pigmentation [37–42].

DEGs (RNA-seq) in black skin vs. white skin were enriched GO terms of melanin biosynthetic process, response to blue light, transmembrane transport, melanosome organization, positive regulation of protein kinase A signalling, developmental pigmentation, melanosome and L-dopa decarboxylase activity. KEGG analysis showed that DEGs were significantly enriched in tyrosine metabolism (4 DEGs), melanogenesis (4 DEGs), neuroactive ligand–receptor interaction (6 DEGs). The result is similar to the previous study [41,42]. Meanwhile, GO terms of m^6^A modified genes also involved in pigmentation, such as regulation of synaptic transmission, dopaminergic, melanosome assembly, regulation of dopamine metabolic process, melanosome organization, negative regulation of kinase activity and melanosome membrane, which is similar to GO enrichment of DEGs. Combined analysis of m^6^A-seq and RNA-seq showed that both the hyper-up genes and hypo-up genes enriched in ErbB signalling pathway which are required to promote normal pigment cell and pigment pattern development in vivo [43].

## Conclusion

In this study, we detected how m^6^A methylation is modiﬁed in black skin and white skin of sheep, and m^6^A modification may play an important role in pigmentation of skin in sheep by participating in AGE-RAGE signalling pathway, ABC transporters, Basal transcription factors and Thyroid hormone synthesis. It provides a basis for further research into the functions of m^6^A methylation modiﬁcations in pigmentation.

## Supplementary Material

Supplemental Material

## Data Availability

All datasets used in this study are available from the corresponding author on reasonable request.

## References

[1] Le L, Sires-Campos J, Raposo G, et al. Melanosome biogenesis in the pigmentation of mammalian skin. Integr Comp Biol. 2021;61(4):1517–15. doi: 10.1093/icb/icab078 34021746 PMC8516112

[2] Ramsden CA, Riley PAJB. Chemistry m. Tyrosinase: the four oxidation states of the active site and their relevance to enzymatic activation, oxidation and inactivation. Bioorg Med Chem. 2014;22(8):2388–2395. doi: 10.1016/j.bmc.2014.02.048 24656803

[3] Boissy RE, Zhao H, Oetting WS, et al. Mutation in and lack of expression of tyrosinase-related protein-1 (TRP-1) in melanocytes from an individual with brown oculocutaneous albinism: a new subtype of albinism classified as “OCA3”. Am J Hum Genet. 1996;58(6):1145–1156. 8651291 PMC1915069

[4] Pennamen P, Tingaud-Sequeira A, Gazova I, et al. Dopachrome tautomerase variants in patients with oculocutaneous albinism. Genet Med. 2021;23(3):479–487. doi: 10.1038/s41436-020-00997-8 33100333

[5] Watt B, van Niel G, Raposo G, et al. PMEL: a pigment cell‐specific model for functional amyloid formation. Pigment Cell Melanoma Res. 2013;26(3):300–315. doi: 10.1111/pcmr.12067 23350640 PMC3633693

[6] Le L, Escobar IE, Ho T, et al. SLC45A2 protein stability and regulation of melanosome pH determine melanocyte pigmentation. Mol Biol Cell. 2020;31(24):2687–2702. doi: 10.1091/mbc.E20-03-0200 32966160 PMC7927184

[7] Zhou S, Zeng H, Huang J, et al. Epigenetic regulation of melanogenesis. Ageing Res Rev. 2021;69:101349. doi: 10.1016/j.arr.2021.101349 33984527

[8] Desrosiers R, Friderici K, Rottman FJPot N. Identification of methylated nucleosides in messenger RNA from Novikoff hepatoma cells. Proc Natl Acad Sci USA. 1974;71(10):3971–3975. doi: 10.1073/pnas.71.10.3971 4372599 PMC434308

[9] Deng X, Chen K, Luo G-Z, et al. Widespread occurrence of N 6-methyladenosine in bacterial mRNA. Nucleic Acids Res. 2015;43(13):6557–6567. doi: 10.1093/nar/gkv596 26068471 PMC4513869

[10] Lence T, Soller M, Roignant J-Y. A fly view on the roles and mechanisms of the m6A mRNA modification and its players. RNA Biol. 2017;14:1232–1240. doi: 10.1080/15476286.2017.1307484 28353398 PMC5699544

[11] Zhao BS, Roundtree IA, CJNrMcb H. Post-transcriptional gene regulation by mRNA modifications. Nat Rev Mol Cell Biol. 2017;18(1):31–42. doi: 10.1038/nrm.2016.132 27808276 PMC5167638

[12] Yue H, Nie X, Yan Z, et al. N6‐methyladenosine regulatory machinery in plants: composition, function and evolution. Plant Biotechnol J. 2019;17(7):1194–1208. doi: 10.1111/pbi.13149 31070865 PMC6576107

[13] Oerum S, Meynier V, Catala M, et al. A comprehensive review of m6A/m6Am RNA methyltransferase structures. Nucleic Acids Res. 2021;49(13):7239–7255. doi: 10.1093/nar/gkab378 34023900 PMC8287941

[14] Zhang J, Yang Q, Yang J, et al. Comprehensive analysis of transcriptome-wide m(6)A methylome upon clostridium perfringens beta2 toxin exposure in porcine intestinal epithelial cells by m(6)A sequencing. Front Genet. 2021;12:689748. doi: 10.3389/fgene.2021.689748 34737761 PMC8560698

[15] Lu Z, Liu J, Yuan C, et al. M(6)a mRNA methylation analysis provides novel insights into heat stress responses in the liver tissue of sheep. Genomics. 2021;113(1):484–492. doi: 10.1016/j.ygeno.2020.09.038 32976974

[16] Dahal U, Le K, Gupta M. RNA m6A methyltransferase METTL3 regulates invasiveness of melanoma cells by matrix metallopeptidase 2. Melanoma Res. 2019;29(4):382–389. doi: 10.1097/CMR.0000000000000580 30762711

[17] Wang ZY, Li P, Hu J, et al. Construction of a single-molecule biosensor for antibody-free detection of locus-specific N 6-methyladenosine in cancer cells and tissues. Anal Chem. 2023;95(12):5454–5462. doi: 10.1021/acs.analchem.3c00730 36930460

[18] Yin L, Ma C, Hou S, et al. Methyltransferase-like (METTL)14-mediated N6-methyladenosine modification modulates retinal pigment epithelial (RPE) activity by regulating the methylation of microtubule-associated protein (MAP)2. Bioengineered. 2022;13(3):4773–4785. doi: 10.1080/21655979.2022.2032968 35139773 PMC8973965

[19] Ma X, Long C, Wang F, et al. METTL3 attenuates proliferative vitreoretinopathy and epithelial-mesenchymal transition of retinal pigment epithelial cells via wnt/β-catenin pathway. J Cell Mol Med. 2021;25(9):4220–4234. doi: 10.1111/jcmm.16476 33759344 PMC8093987

[20] Zha X, Xi X, Fan X, et al. Overexpression of METTL3 attenuates high-glucose induced RPE cell pyroptosis by regulating miR-25-3p/PTEN/Akt signaling cascade through DGCR8. Aging. 2020;12(9):8137. doi: 10.18632/aging.103130 32365051 PMC7244028

[21] Zeng Y, Wang S, Gao S, et al. Refined RIP-seq protocol for epitranscriptome analysis with low input materials. PLoS Biol. 2018;16(9):e2006092. doi: 10.1371/journal.pbio.2006092 30212448 PMC6136692

[22] Bolger AM, Lohse M, Usadel BJB. Trimmomatic: a flexible trimmer for Illumina sequence data. Bioinformatics. 2014;30(15):2114–2120. doi: 10.1093/bioinformatics/btu170 24695404 PMC4103590

[23] Yang S, Fan R, Shi Z, et al. Identification of a novel microRNA important for melanogenesis in alpaca (Vicugna pacos). J Anim Sci. 2015;93(4):1622–1631. doi: 10.2527/jas.2014-8404 26020184

[24] He S, Wang H, Liu R, et al. mRNA N6-methyladenosine methylation of postnatal liver development in pig. PLoS One. 2017;12(3):e0173421. doi: 10.1371/journal.pone.0173421 28267806 PMC5340393

[25] Dominissini D, Moshitch-Moshkovitz S, Schwartz S, et al. Topology of the human and mouse m6A RNA methylomes revealed by m6A-seq. Nature. 2012;485(7397):201–206. doi: 10.1038/nature11112 22575960

[26] Qin Z, Wang W, Ali MA, et al. Transcriptome-wide m(6)A profiling reveals mRNA post-transcriptional modification of boar sperm during cryopreservation. BMC Genomics. 2021;22(1):588. doi: 10.1186/s12864-021-07904-8 34344298 PMC8335898

[27] Meyer KD, Saletore Y, Zumbo P, et al. Comprehensive analysis of mRNA methylation reveals enrichment in 3'UTRs and near stop codons. Cell. 2012;149(7):1635–1646. doi: 10.1016/j.cell.2012.05.003 22608085 PMC3383396

[28] Price MA, Colvin Wanshura LE, Yang J, et al. CSPG4, a potential therapeutic target, facilitates malignant progression of melanoma. Pigment Cell Melanoma Res. 2011;24(6):1148–1157. doi: 10.1111/j.1755-148X.2011.00929.x 22004131 PMC3426219

[29] Zhao-Qi ZHOU, Khan A, Qiong JIA, et al. TMEM106B induces melanogenesis by regulating ERK/CREB signaling pathway. Chinese J Biochem Mol Biol. 2021;37(10):1386–1393.

[30] Bevitt DJ, Mohamed J, Catterall JB, et al. Expression of ADAMTS metalloproteinases in the retinal pigment epithelium derived cell line ARPE-19: transcriptional regulation by TNFα. Biochim Biophys Acta. 2003;1626(1–3):83–91. doi: 10.1016/S0167-4781(03)00047-2 12697333

[31] Kociok N, Joussen A, Ophthalmology E. Varied expression of functionally important genes of RPE and choroid in the macula and in the periphery of normal human eyes. Graefe’s Arch Clin Exp Ophthalmol. 2007;245(1):101–113. doi: 10.1007/s00417-006-0266-x 16598467

[32] Chernoff KA, Bordone L, Horst B, et al. GAB2 amplifications refine molecular classification of melanoma. Clin Cancer Res. 2009;15(13):4288–4291. doi: 10.1158/1078-0432.CCR-09-0280 19509136 PMC2878201

[33] Nishida K, Wang L, Morii E, et al. Requirement of Gab2 for mast cell development and KitL/c-Kit signaling. Blood. 2002;99(5):1866–1869. doi: 10.1182/blood.V99.5.1866 11861309

[34] D’Mello SA, Finlay GJ, Baguley BC, et al. Signaling pathways in melanogenesis. Int J Mol Sci. 2016;17:1144. doi: 10.3390/ijms17071144 27428965 PMC4964517

[35] Onishi H, Ichimiya S, Yanai K, et al. RBPJ and MAML3: potential therapeutic targets for small cell lung cancer. Anticancer Res. 2018;38(8):4543–4547. doi: 10.21873/anticanres.12758 30061220

[36] Bushby K, Collins J, Hicks DH. Collagen type VI myopathies. Adv Exp Med Biol. 2014;12:185–199. 10.1007/978-94-007-7893-1_12 24443028

[37] Lu Y, Bowswell M, Bowswell W, et al. Molecular genetic response of Xiphophorus maculatus–X. couchianus interspecies hybrid skin to UVB exposure. Comp Biochem Physiol C Toxicol Pharmacol. 2015;178:86–92. doi: 10.1016/j.cbpc.2015.07.011 26254713 PMC4662913

[38] Jia Q, Hu S, Jiao D, et al. Synaptotagmin-4 promotes dendrite extension and melanogenesis in alpaca melanocytes by regulating Ca 2+ influx via TRPM1 channels. Cell Biochem Funct. 2020;38(3):275–282. doi: 10.1002/cbf.3465 31743468 PMC7318172

[39] Villarreal-Reyna G, Garza-Morales R, Soto-Domínguez A, et al. Cerebrolysin induces hair repigmentation associated to MART-1/Melan-A reactivation. Eur J Med Res. 2022;27(1):1–8. doi: 10.1186/s40001-022-00889-4 36411485 PMC9677656

[40] Abolins‐Abols M, Kornobis E, Ribeca P, et al. Differential gene regulation underlies variation in melanic plumage coloration in the dark-eyed junco (Junco hyemalis). Mol Ecol. 2018;27(22):4501–4515. doi: 10.1111/mec.14878 30252177

[41] Fan R, Xie J, Bai J, et al. Skin transcriptome profiles associated with coat color in sheep. BMC Genomics. 2013;14(1):1–12. doi: 10.1186/1471-2164-14-389 23758853 PMC3689618

[42] Shi X, Wu J, Lang X, et al. Comparative transcriptome and histological analyses provide insights into the skin pigmentation in Minxian black fur sheep (Ovis aries). PeerJ. 2021;9:e11122. doi: 10.7717/peerj.11122 33986980 PMC8086576

[43] Budi EH, Patterson LB, Parichy DM. Embryonic requirements for ErbB signaling in neural crest development and adult pigment pattern formation. Development. 2008;135(15):2603–2614. doi: 10.1242/dev.019299 18508863 PMC2704560

